# Relaxation Along a Fictitious Field, continuous wave T1rho, adiabatic T1rho and adiabatic T2rho imaging of human gliomas at 3T: A feasibility study

**DOI:** 10.1371/journal.pone.0296958

**Published:** 2024-04-01

**Authors:** Ivan Jambor, Aida Steiner, Marko Pesola, Maria Gardberg, Janek Frantzén, Pekka Jokinen, Timo Liimatainen, Heikki Minn, Hannu Aronen, Harri Merisaari

**Affiliations:** 1 Department of Radiology, University of Turku, Turku, Finland; 2 Enterprise Service Group—Radiology, Mass General Brigham, Boston, MA, United States of America; 3 Medical Imaging Centre of Southwest Finland, Turku University Hospital, Turku, Finland; 4 Tyks Laboratories, Pathology, Turku University Hospital and Institute of Biomedicine, University of Turku Turku, Finland; 5 Division of Clinical Neurosciences, Department of Neurosurgery, Turku University Hospital and University of Turku, Turku, Finland; 6 Department of Radiology, University of Oulu, Oulu, Finland; 7 Department of Biotechnology and Molecular Medicine, A.I. Virtanen Institute for Molecular Sciences, University of Eastern Finland, Kuopio, Finland; 8 Department of Oncology and Radiotherapy, Turku University Hospital, Turku, Finland; 9 Turku PET Centre, Turku University and Turku University Hospital, Turku, Finland, Finland; University of Michigan Medical School, UNITED STATES

## Abstract

In pre-clinical models of brain gliomas, Relaxation Along a Fictitious Field in second rotating frame (T_RAFF2_), continues wave T_1rho_ (T_1ρcw_), adiabatic T_1rho_ (T_1ρadiab_), and adiabatic T_2rho_ (T_2ρadiab_) relaxation time mappings have demonstrated potential to non-invasively characterize brain gliomas. Our aim was to evaluate the feasibility and potential of 4 different spin lock methods at 3T to characterize primary brain glioma. 22 patients (26–72 years) with suspected primary glioma. T_1ρcw_ was performed using pulse peak amplitude of 500Hz and pulse train durations of 40 and 80 ms while the corresponding values for T_1ρadiab_, T_2ρadiab_, T_RAFF2_ were 500/500/500Hz and 48 and 96, 64 and 112, 45 and 90 ms, respectively. The parametric maps were calculated using a monoexponential model. Molecular profiles were evaluated from tissue specimens obtained during the resection. The lesion regions-of-interest were segmented from high intensity FLAIR using automatic segmentation with manual refinement. Statistical descriptors from the voxel intensity values inside each lesion and radiomic features (Pyrad MRC package) were calculated. From extracted radiomics, mRMRe R package version 2.1.0 was used to select 3 features in each modality for statistical comparisons. Of the 22 patients, 10 were found to have IDH-mutant gliomas and of those 5 patients had 1p/19q codeletion group comparisons. Following correction for effects of age and gender, at least one statistical descriptor was able to differentiate between IDH and 1p/19q codeletion status for all the parametric maps. In the radiomic analysis, corner-edge detector features with Harris-Stephens filtered signal showed significant group differences in IDH and 1p/19q codeletion groups. Spin lock imaging at 3T of human glioma was feasible and various qualitative parameters derived from the parametric maps were found to have potential to differentiate IDH and 1p19q codeletion status. Future larger prospective clinical trials are warranted to evaluate these methods further.

## Introduction

Gliomas are the most common primary brain neoplasms. They range from low grade to anaplastic gliomas and glioblastoma. In the most recent 2021 WHO classification of central nervous system tumors, astrocytic gliomas and oligodendrogliomas are primarily differentiated based on molecular markers [1]. IDH mutation and 1p/19q codeletion are the two most important markers predicting longer survival and distinguishing oligodendrogliomas from other diffuse astrocytomas, respectively. The current standard criterion for tumor grading is based on the combined histological and molecular diagnosis of tumor specimen obtained during surgery. However, this may have limitations due to sampling errors especially when tumor-grade heterogeneity is present within the tumor. These sampling errors could have consequences on the management of the disease and survival. Therefore, novel more accurate non-invasive imaging tools which could predict cancer aggressiveness might complement the integrated molecular and histopathologic grade [2].

Relaxation along a fictitious field (RAFF) is an MRI technique applying amplitude and frequency-modulated irradiation in a sub-adiabatic regime [3]. The use of radiofrequency pulse is based on sine and cosine amplitude and frequency modulations of equal amplitudes, which give rise to a stationary fictitious magnetic field in a doubly rotating frame. The RAFF relaxation time constant was found to differ from laboratory frame relaxation times (T_1_ and T_2_) and rotating frame relaxation times, such as continues wave T_1rho_ (T_1ρcw_), adiabatic T_1rho_ (T_1ρadiab_), and adiabatic T_2rho_ (T_2ρadiab_) [4]. Rotating frame relaxations have shown to be quantitative MRI markers to follow up disease progression, including brain ischemia [5]. Moreover, RAFF relaxation values have shown excellent correlation with cell density in a rat glioma model, which makes it a potential biomarker to follow up cancer therapy outcome [6].

Our hypothesis is that improved preoperative characterization delineation of brain tumors using novel MRI techniques, including spin lock imaging could provide tools for better treatment selection. Thus, the purpose of this study was to evaluate the feasibility of spin locking methods performed using Relaxation Along a Fictitious Field in second rotating frame (T_RAFF2_), continuous wave T_1rho_ (T_1ρcw_), adiabatic T_1rho_ (T_1ρadiab_), and adiabatic T_2rho_ (T_2ρadiab_) and their potential to non-invasively characterize brain glioma.

## Material and methods

### Study design and study population

The study was approved by the local ethics committee (Turku University Hospital, Turku, Finland) and the ClinicalTrials.gov Identifier is NCT02186262. Each patient with suspected primary glioma gave written inform consent.

### MRI protocol and MRI reporting

The MRI examinations were performed using 3T Philips system (Ingenuity PET/MR, Philips, Cleveland, OH). Two channel volume whole body RF coil was used for the excitation while 32 channel manufacture’s head coil was used as a receiver coil. All four spin locking imaging methods, T_RAFF2_, T_1ρcw_, T_1ρadiab_, and T_2rho_,Table were measured using 3D T1-FFE sequence. The spin lock weightings were repeated once in every 3 s with 20 acquisitions after each weighting. Other parameters were: repetition time of individual acquisitions/echo time (TR/TE) 4.9/2.4 ms, acquisition voxel size 1.25x1.84x2.50 mm^3^, reconstruction voxel size 1.25x1.25x2.50 mm^3^, 24 slices, centric k-space coding, acquisition time 2 minutes 15 seconds per each frame. T_1ρcw_ was performed using a block pulse with radiofrequency (RF) peak amplitude 500 Hz (corresponding to 11.74 mT, B_1_) and pulse durations of 40 and 80 ms. T1_ρadiab_ was performed using hyperbolic secant pulses with RF peak amplitude 500 Hz (corresponding to 11.74 mT, B_1_) and pulse durations of 48 and 96 ms. The pulse train duration was 48 ms and 96 ms. T_RAFF2_ was performed with RF peak amplitude of 500 Hz (corresponding to 11.74 mT, B_1_) and pulse train duration of RAFF2 68 ms and 135 ms. In addition, FLAIR-weighted images were acquired using a 3D TSE sequence with the following parameters: TR/TE 8000/337 ms, inversion time 2400 ms, acquisition voxel size with whole brain coverage 1.0x1.0x2.0 mm^3^, reconstruction voxel size with whole brain coverage 1.0x1.0x1.0 mm^3^, FOV 256x256 mm^2^, SENSE factor 2.0 in right-left and 2.0 and anterior-posterior direction, acquisition time 5 minutes 28 seconds. All MRI examinations were performed within specific absorption rate limit.

### Histology and immunohistochemistry

All histopathological material was reviewed by one of the co-authors (MG), board certified pathologist since 2008 with neuropathologist subspecialty since 2011, with 15 years of experience in neuropathology. The histological and molecular pathology was performed at Tyks Laboratories, Turku, Finland, which is accredited by the Finnish Accreditation services, according to the ISO standard 15189:2013. Histology of brain lesions is within the accreditation field, and thus quality control is well documented. Tumor tissue samples were formalin-fixed and paraffin embedded (FFPE). FFPE sections were H&E stained, and immunohistochemistry was performed on a Ventana Benchmark XT Autostainer (Ventana, Strasbourg, France) using an anti-IDH1 R132H antibody at 1:50 dilution (clone H09, Dianova, Hamburg, Germany). Tumors negative for an immunohistochemically detectable IDH1 mutation were genetically tested for IDH1/IDH2 mutations. All tumors were tested for 1p19q codeletion by fluorescent *in situ* hybridization using Vysis 1p36/1q25 and 19q13/19p13 FISH probe kit (Abbot Laboratories, Abbot Park, IL). Two diffuse astrocytic gliomas without IDH1/2 mutations were further tested for EGFR amplification using silver in situ hybridization. With all stainings and molecular information available, an integrated diagnosis according to the current WHO classification.

For Ki-67 staining, a rabbit monoclonal antibody (clone 30–9, Ventana) was used. The proliferation index was evaluated in the most highly proliferating hotspot area by counting positively stained nuclei/all nuclei in a high-power microscopic field (40x objective).

### Data analysis

The relaxation time values T_RAFF2_, T_1ρcw_, T_1ρadiab_, and T_2ρadiab_ were calculated using two parameter monoexponential model. Rigid co-registration was performed using FSL [7] 5.0.4 so all modalities were aligned to the FLAIR images. The lesion ROIs (Region of Interest) were segmented from FLAIR images using automatic segmentation procedure described by Artzi [8]. Tumor region was defined manually from segmented ROIs by applying 1–2 voxel erosion after the automatic procedure.

Voxel level parametric maps of the fitted relaxation values were analyzed by calculating statistical descriptors from the voxel intensity values inside each lesion ROI: 10th, 25th, 50th, 75th, and 90th percentile; kurtosity; skewness; median. For radiomics, we applied radiomic feature extraction using Pyrad package [9] and MRC package [10]. From extracted radiomics, mRMRe R package version 2.1.0 [11] was used to select 3 features in each modality for statistical comparisons.

### Statistical analysis

Student’s unpaired t-test was applied for comparison between groups of IDH-mutant vs IDH-wildtype, and astrocytic gliomas without 1p/19q codeletion vs oligodendrogliomas with 1p/19q codeletion, and Pearson’s r for correlation between relaxation values and Ki-67 index. All statistical tests were done in RStudio environment (v 1.1.383, 2017 RStudio, Inc.). FDR-corrected p-values smaller than 0.05 were considered statistically significant, unless otherwise noted.

## Results

**Fig 1:**
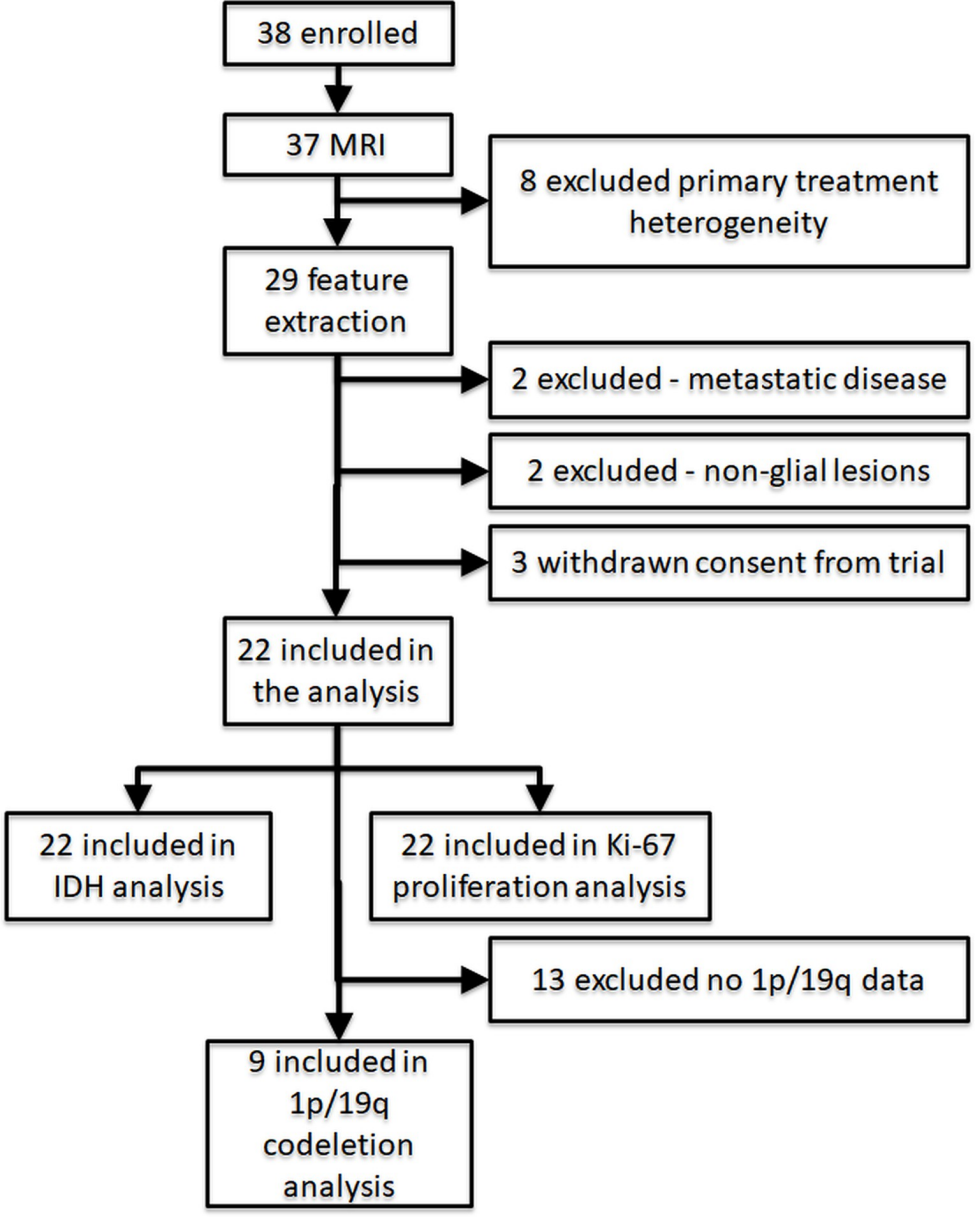
Patient selection flow diagram for evaluations.

In total, 38 patients were enrolled between 02/2014 and 9/2015, of those 37 underwent the brain MRI examination. Due to slow enrolment, also patients with suspected recurrent gliomas were enrolled (n = 8) but these patients were not included in the final analyses due to large heterogeneity of primary treatment between these cases. In the remaining 29 patients, 2 were found to have metastatic disease following surgical resection, 2 were found to have non-glial lesions (one meningioma, one with epidermoid), and 3 withdrawn from the trial precluding the use of histopathological material for the analyses in this trial. Thus, 22 patients were included in the final IDH group and Ki-67 proliferation index analysis, and 9 patients in 1p/19q codeletion group comparisons (Fig 1).

**Fig 2:**
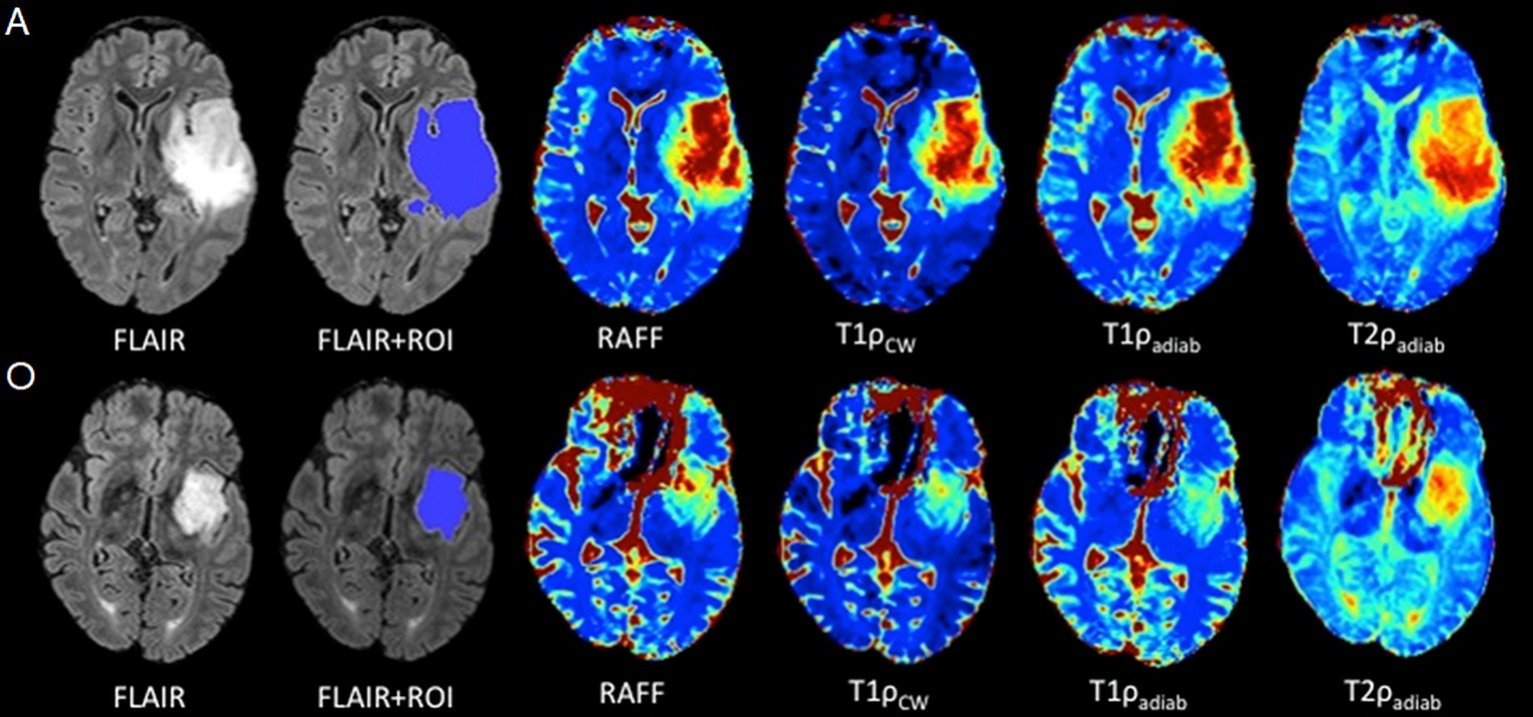
Two representative cases: 1: Diffuse astrocytoma IDH-mutant, grade 2 (A), (upper row) and 2: oligodendroglioma, 1p/19q codeleted, grade 2 (O) (bottom row) demonstrate automatic FLAIR lesion segmentation and imaging contrasts for T_RAFF2_, T_1ρcw_, T_1ρadiab_, and T_2ρadiab_ parametric maps. Same scaling was applied within the same sequence to allow comparison between the gliomas. Diffuse astrocytoma, IDH-mutant shows elevated values in all parametric maps compared to oligodendroglioma, IDH-mutant and 1p/19q codeleted. This trend was observed across the cohort, however, without statistical significance.

Visual inspection of T_RAFF2_, T_1ρcw_, T_1ρadiab_, and T_2ρadiab_ demonstrated good tissue contrast between glioma and normal brain tissue as presented in Fig 2.

In the group comparisons, at least one statistical descriptor per each spin lock imaging method was able to differentiate between IDH and 1p/19q codeletion groups after correcting for age and gender (Tables 1 and 2, S1 and S2 Tables in S1 File), however, no statistically significant difference was found with ROI mean before correction for effects of age and gender. In the ROI based analysis, only FLAIR derived variables were found to have significant correlation with Ki-67 (Table 3, S3 Table in S1 File).

**Table 1:** Region of Interest (ROI) mean intensity differences between IDH-mutated (IDH mut) vs IDH-wild-type (IDH wt) gliomas. Model Variable~Group+Age+Gender-1 was used to correct for effects of age and gender.

Modality	MRI Variable	IDH all	IDH mut	IDH wild type	FDR-corrected p-value
T2W	skewness	0.77±1.08	0.79±1.03	0.75±1.14	<0.001
	kurtosis	2.97±4.84	2.37±3.53	3.56±5.82	<0.001
T2HS	mean	51.73±29.82	51.08±29.41	52.37±30.57	<0.001
	median	50.93±33.58	51.13±34.39	50.72±33.18	<0.001
	75percentile	62.95±36.67	61.71±33.92	64.16±39.55	<0.001
	skewness	0.66±4.40	1.33±5.91	0.00±1.92	<0.001
	kurtosis	20.13±111.29	37.32±157.17	3.35±9.28	<0.001
	SD	20.31±15.03	18.57±13.56	22.02±16.32	<0.001
T1HS	mean	189.11±149.76	159.12±101.16	218.39±181.93	<0.001
	median	168.33±153.41	147.58±101.92	188.58±189.97	<0.001
	25percentile	119.08±119.30	104.90±95.25	132.92±138.62	<0.001
	75percentile	232.57±216.45	189.40±117.97	274.72±276.48	<0.001
	skewness	1.76±2.87	2.31±3.44	1.23±2.08	<0.001
	kurtosis	13.68±42.70	20.99±59.22	6.54±10.90	<0.001
	SD	112.93±99.94	87.18±70.54	138.07±117.52	<0.001
T1CW	mean	84.74±67.91	73.99±52.69	95.24±79.28	<0.001
	median	64.89±47.68	66.16±47.31	63.65±48.59	0.01
	75percentile	93.27±78.22	86.29±60.51	100.09±92.57	<0.001
	kurtosis	26.21±52.56	35.58±67.90	17.06±29.23	0.02
	SD	75.91±90.09	51.56±54.60	99.67±110.24	<0.001
RAFF	mean	141.58±113.01	129.70±92.59	153.17±130.01	<0.001
	median	118.57±104.35	116.85±83.14	120.26±122.59	<0.001
	25percentile	83.84±81.61	81.60±73.40	86.02±89.76	<0.001
	75percentile	167.85±161.89	154.21±109.21	181.16±201.04	<0.001
	kurtosis	18.63±47.71	26.36±65.01	11.08±17.60	<0.001
	SD	100.35±98.59	79.64±73.29	120.57±115.55	<0.001
FLAIR	25percentile	1024.05±274.49	1105.59±265.59	944.45±262.16	0.02
	skewness	1.00±1.19	0.89±1.07	1.11±1.30	<0.001
f2000	mean	0.21±0.13	0.19±0.13	0.22±0.13	<0.001
	median	0.19±0.15	0.18±0.14	0.19±0.15	<0.001
	25percentile	0.12±0.12	0.11±0.12	0.13±0.11	<0.001
	75percentile	0.27±0.17	0.25±0.16	0.28±0.17	<0.001
	kurtosis	20.45±121.18	31.24±168.58	9.93±37.58	<0.001
	SD	0.14±0.09	0.12±0.07	0.16±0.10	<0.001
Ds2000	mean	0.02±0.04	0.01±0.04	0.03±0.05	<0.001
	SD	0.07±0.11	0.06±0.09	0.08±0.12	<0.001
Ds4000	mean	0.02±0.04	0.01±0.04	0.03±0.05	<0.001
	25percentile	0.00±0.00	0.00±0.00	0.00±0.00	0.02
	SD	0.07±0.11	0.06±0.09	0.08±0.12	<0.001
Df2000	mean	0.05±0.10	0.03±0.06	0.06±0.12	<0.001
	median	0.03±0.11	0.02±0.04	0.05±0.15	<0.001
	25percentile	0.01±0.04	0.01±0.02	0.01±0.05	<0.001
	75percentile	0.06±0.14	0.04±0.08	0.09±0.18	<0.001
	SD	0.06±0.09	0.04±0.06	0.07±0.10	<0.001
Df4000	mean	0.14±0.16	0.12±0.15	0.15±0.17	<0.01
	median	0.11±0.16	0.11±0.16	0.12±0.17	0.05
	25percentile	0.05±0.12	0.05±0.09	0.06±0.14	<0.001
	75percentile	0.19±0.25	0.17±0.23	0.22±0.27	0.01
	kurtosis	25.18±124.26	34.81±168.35	15.78±55.17	<0.001
	SD	0.11±0.11	0.10±0.10	0.13±0.12	<0.001

**Table 2:** Region of Interest (ROI) mean intensity differences between astrocytic gliomas without 1p/19q codeletion (A) v oligodendroglioma IDH-mutant and 1p/19q codeleted (O). Model Variable~Group+Age+Gender-1 model was used to correct for effects of age and gender.

Modality	Statistic	All	A	O	FDR-corrected -value
T2W	mean	1025.79±182.84	1023.76±180.65	1026.73±187.38	<0.01
	median	1012.22±196.09	998.50±182.84	1018.55±205.11	<0.01
	25percentile	913.07±133.86	899.47±132.46	919.34±136.63	<0.01
	75percentile	1123.49±255.58	1127.97±243.63	1121.43±265.60	<0.01
	skewness	0.79±1.00	0.78±0.81	0.80±1.08	<0.001
	kurtosis	2.14±3.33	1.39±2.84	2.48±3.54	<0.001
T2HS	mean	49.77±29.95	47.10±28.57	51.00±31.04	<0.001
	median	49.43±35.14	47.82±34.97	50.17±35.88	<0.001
	25percentile	38.20±34.24	33.45±34.15	40.39±34.72	<0.001
	75percentile	60.55±34.96	58.75±35.77	61.38±35.26	<0.001
	SD	18.87±13.51	21.31±14.17	17.74±13.33	<0.001
T1HS	mean	155.61±103.68	153.86±98.44	156.42±107.90	<0.01
	median	142.82±104.05	139.11±104.82	144.54±105.73	<0.001
	25percentile	102.48±94.66	92.77±97.31	106.96±95.02	<0.001
	75percentile	186.19±121.52	187.41±121.84	185.64±123.78	<0.01
	skewness	2.30±3.56	2.10±2.00	2.39±4.12	<0.01
	kurtosis	21.87±61.46	13.98±19.80	25.50±73.31	<0.01
T1CW	median	64.75±48.81	58.67±44.05	67.56±51.44	<0.01
	25percentile	45.81±42.13	38.98±40.27	48.96±43.36	<0.001
	75percentile	85.90±62.80	77.62±49.75	89.72±68.56	0.03
	skewness	3.18±3.93	3.01±2.53	3.25±4.47	<0.001
	kurtosis	36.15±70.36	31.13±58.98	38.47±76.02	<0.001
RAFF	median	114.28±85.59	106.66±81.74	117.80±88.66	<0.01
	25percentile	80.13±73.32	71.02±73.92	84.34±74.12	<0.001
	75percentile	153.42±113.18	141.86±93.06	158.76±122.69	0.03
	skewness	2.60±3.92	2.57±2.13	2.60±4.56	<0.001
	kurtosis	26.20±67.31	18.71±27.24	29.67±79.62	<0.001
FLAIR	mean	1256.28±302.95	1260.09±340.42	1254.53±291.26	<0.001
	median	1229.55±306.88	1233.05±333.85	1227.93±300.55	<0.001
	25percentile	1114.85±273.84	1103.11±287.21	1120.26±273.11	<0.001
	75percentile	1373.31±353.19	1389.10±397.00	1366.01±339.26	<0.001
	skewness	0.69±0.51	0.58±0.39	0.74±0.56	<0.001
	kurtosis	1.06±1.63	0.38±0.85	1.37±1.81	<0.001
f2000	mean	0.19±0.13	0.19±0.11	0.20±0.14	0.03
	median	0.18±0.15	0.17±0.13	0.18±0.15	0.02
	75percentile	0.25±0.17	0.23±0.14	0.26±0.18	<0.01
	skewness	2.10±5.37	1.38±1.53	2.43±6.43	<0.01
	SD	0.13±0.08	0.13±0.08	0.13±0.07	<0.01
Ds2000	median	0.00±0.00	0.00±0.00	0.00±0.00	<0.01
	25percentile	0.00±0.00	0.00±0.00	0.00±0.00	0.03
	75percentile	0.00±0.00	0.00±0.00	0.00±0.00	<0.01
	skewness	4.73±8.74	1.51±2.89	6.21±10.11	<0.001
	kurtosis	95.39±284.84	8.87±13.36	135.33±338.74	<0.01
Ds4000	median	0.00±0.00	0.00±0.00	0.00±0.00	<0.001
	25percentile	0.00±0.00	0.00±0.00	0.00±0.00	<0.001
	75percentile	0.00±0.00	0.00±0.00	0.00±0.00	<0.001
	skewness	4.57±8.83	1.34±2.93	6.06±10.21	<0.001
	kurtosis	95.58±284.78	8.54±13.46	135.76±338.58	<0.01
Df2000	skewness	4.47±5.72	5.04±4.05	4.21±6.41	<0.01
Df4000	skewness	2.66±5.32	2.44±1.55	2.77±6.39	<0.01

**Table 3:** Pearson correlation analysis between Ki-67 index and MRI relaxation time constants in brain glioma patients, using median value inside (N = 22. N(lesions) = 83). Age and Gender were evaluated with Pearson correlation, while other MRI variables were evaluated with model Ki67~Variable_MRI VARIABLE_+Age+Gender-1. Only Age and FLAIR were found to have statistically significant correlation after correction for multiple comparisons over evaluations on basic statistical descriptors.

**MRI Variable**	**Pearson r**	**p-value**	**FDR-corrected p-value**
Age	0.367	<0.001	<0.001
Gender	-0.039	0.726	0.920
**MRI Variable**	**beta**	**p-value**	**FDR-corrected p-value**
FLAIR mean	-0.338	0.002	0.038
FLAIR median	-0.346	0.001	0.032
FLAIR 25percentile	-0.387	<0.001	0.012

In the radiomic analyses, corner-edge detector features with Harris-Stephens filtered signal were selected in feature selection step for most of the modalities, and also showed significant group differences in IDH and 1p/19q codeletion evaluations (Tables 4 and 5). Various features prominently from pyradiomics package were selected and demonstrated significant correlation with Ki-67 correlations (Table 6).

**Table 4:** Region of Interest (ROI) radiomic feature differences between IDH-mutated (IDH mut) vs IDH-wild-type (IDH wt) gliomas, for features after feature selection. P-values are fdr-corrected 110 over evaluated radiomics. For MRI variables Group_IDH_~Variable_RADIOMIC_+Age+Gender-1 model was used to correct for effects of age and gender.

Modality	Radiomic Feature	FDR-corrected p-value
T2W	FFT FWHM 2.00 LP	0.001
	FFT FWHM 4.00 LP	0.001
	FFT FWHM 1.00 LP	0.002
T2HS	H-S b2 ks3 k0.05 CED ratio overall	<0.001
	H-S b3 ks3 k0.01 CED ratio overall	<0.001
	H-S b2 ks1 k0.01 CED ratio overall	<0.001
T1HS	H-S b3 ks1 k0.05 CED ratio overall	<0.001
	H-S b3 ks3 k0.01 CED ratio overall	<0.001
	H-S b2 ks1 k0.01 CED ratio overall	<0.001
T1CW	H-S b2 ks1 k0.05 CED ratio	0.002
	H-S b3 ks7 k0.05 CED ratio overall	0.002
	H-S b2 ks1 k0.01 CED ratio overall	0.002
RAFF	H-S b2 ks1 k0.05 CED ratio	0.001
	H-S b2 ks7 k0.01 CED ratio overall	0.001
	H-S b2 ks1 k0.01 CED ratio overall	0.001
FLAIR	H-S b2 ks3 k0.01 CED ratio	<0.001
	H-S b3 ks1 k0.05 CED ratio overall	<0.001
	H-S b2 ks1 k0.01 CED ratio overall	<0.001
f2000	H-S b2 ks3 k0.01 CED ratio	0.031
	H-S b2 ks7 k0.01 CED ratio overall	0.002
	H-S b2 ks1 k0.01 CED ratio overall	0.009
Ds2000	H-S b2 ks1 k0.05 No corners ratio	0.001
	H-S b3 ks1 k0.05 CED mean	0.003
	H-S b2 ks1 k0.01 CED ratio overall	0.031
Ds4000	H-S b2 ks1 k0.05 No corners ratio	<0.001
	H-S b3 ks3 k0.05 CED ratio	0.056
	H-S b2 ks1 k0.01 CED ratio overall	0.051
Df2000	H-S b2 ks3 k0.01 CED ratio	0.013
	H-S b3 ks1 k0.05 CED ratio overall	0.011
	H-S b2 ks1 k0.01 CED ratio overall	0.001
Df4000	H-S b2 ks3 k0.01 CED ratio	0.044
	H-S b3 ks3 k0.01 CED ratio overall	0.018
	H-S b2 ks1 k0.01 CED ratio overall	0.002

**FFT:** 2D Fast Fourer Transform (FWHM = Smoothing Full Width Half Maximum in mm, LP = Low Pass filtered)

**H-S:** Harris-Stephens filter based radiomic with corresponding parameters

**CED ratio:** Corner-edge location density ratio between ROI and white matter over slices containing lesion

**CED ratio overall:** Corner-edge location density ratio between ROI and white matter over all slices

**No corners:** Number of detected corner-edge locations

**Table 5:** Region of Interest (ROI) radiomic feature differences between astrocytic gliomas without 1p/19q codeletion (A) v oligodendroglioma IDH-mutant and 1p/19q codeleted (O), for features after feature selection. P-values are fdr-corrected over 110 evaluated radiomics. For MRI variables Group_IDH_~Variable_RADIOMIC_+Age+Gender-1 model was used to correct for effects of age and gender.

Modality	Radiomic Feature	FDR-corrected p-value
T2W	FFT rel f1.0 FWHM2.00 LP	0.026
	Gabor f1.50 d2 k2 Cmean	0.224
	FFT rel f1.0 FWHM1.00 LP	0.026
T2HS	H-S b3 ks1 k0.05 CED mean	0.001
	H-S b2 ks7 k0.50 CED ratio overall	<0.001
	H-S b2 ks1 k0.01 CED ratio overall	<0.001
T1HS	H-S b3 ks1 k0.05 CED ratio overall	<0.001
	H-S b3 ks3 k0.01 CED ratio overall	<0.001
	H-S b2 ks1 k0.01 CED ratio overall	<0.001
T1CW	H-S b2 ks7 k0.01 CED ratio overall	<0.001
	H-S b3 ks7 k0.05 CED ratio overall	<0.001
	H-S b2 ks1 k0.01 CED ratio overall	<0.001
RAFF	H-S b3 ks1 k0.05 CED primary	<0.001
	H-S b2 ks7 k0.01 CED ratio overall	<0.001
	H-S b2 ks1 k0.01 CED ratio overall	<0.001
FLAIR	H-S b2 ks1 k0.05 CED ratio	<0.001
	H-S b2 ks7 k0.50 CED mean	<0.001
	H-S b2 ks1 k0.01 CED ratio overall	<0.001
f2000	H-S b3 ks3 k0.01 CED ratio overall	<0.001
	H-S b2 ks7 k0.01 CED ratio overall	<0.001
	H-S b2 ks1 k0.01 CED ratio overall	<0.001
Ds2000	H-S b2 ks3 k0.05 CED ratio overall	0.327
	H-S b3 ks7 k0.50 CED secondary	0.001
	H-S b2 ks1 k0.01 CED ratio overall	0.126
Ds4000	H-S b2 ks1 k0.50 CED ratio overall	0.126
	H-S b3 ks7 k0.05 CED ratio overall	0.001
	H-S b2 ks1 k0.01 CED ratio overall	0.599
Df2000	H-S b2 ks3 k0.01 CED secondary	0.199
	H-S b3 ks3 k0.01 CED ratio overall	0.001
	H-S b2 ks1 k0.01 CED ratio overall	<0.001
Df4000	H-S b3 ks7 k0.05 CED mean	0.007
	H-S b3 ks1 k0.05 CED ratio overall	0.001
	H-S b2 ks1 k0.01 CED ratio overall	<0.001

**FFT:** 2D Fast Fourer Transform (FWHM = Smoothing Full Width Half Maximum in mm, LP = Low Pass filtered)

**Gabor**: Gabor filtered blob mean value

**H-S:** Harris-Stephens filter based radiomic with corresponding parameters

**CED ratio:** Corner-edge location density ratio between ROI and white matter over slices containing lesion

**CED ratio overall:** Corner-edge location density ratio between ROI and white matter over all slices

**No corners:** Number of detected corner-edge locations

**CED primary:** Corner-edge density distribution primary extent

**CED secondary:** Corner-edge density distribution secondary extent

**CED mean:** Corner-edge density distribution mean

**Table 6:** Pearson correlation analysis between Ki-67 index and MRI relaxation time constants in brain glioma patients, for features after feature selection. The radiomic feature values were evaluated with model Ki67~Variable_RADIOMIC FEATURE_ +Age+Gender-1.

Modality	Radiomic Feature	FDR-corrected p-value
T2W	**Pyrad wavelet_LLH_glcm_Correlation**	**0.005**
	**Pyrad_log_sigma_1mm_3D Uniformity**	**0.009**
	**Pyrad wavelet_HLL_glcm_JointEntropy**	**0.032**
T2HS	Pyrad wavelet_LLH_glszm_SmallAreaHighGrayLevelEmphasis	0,093
	**Pyrad_log_sigma_3mm_3D_glszm_SizeZoneNonUniformity**	**<0.001**
	Pyrad_log_sigma_1mm_3D_glszm_SmallAreaHighGrayLevelEmphasis	0,211
T1HS	**Frangi_objprops_Per_mean_mm**	**<0.001**
	Pyrad InterquartileRange	0,185
	**Frangi_objprops_Rel_ecc**	**0.006**
T1CW	**Pyrad_log_sigma_3mm_3D_glszm_SmallAreaHighGrayLevelEmphasis**	**<0.001**
	Pyrad_log_sigma_3mm_3D_glcm_Contrast	0.069
	**Pyrad_wavelet_HHL_glszm_SmallAreaHighGrayLevelEmphasis**	**0.001**
RAFF	**Gabor_f1.00_d2_k16_skewness**	**<0.001**
	**Pyrad_log_sigma_3mm_3D_glszm_SmallAreaHighGrayLevelEmphasis**	**<0.001**
	Pyrad_log_sigma_3mm_3D_glcm_Contrast	0,090
FLAIR	**Hessian_0.005_15.0_objprops_Relative_2**^**nd**^ **axis length**	**0.034**
	**Hessian_0.025_15.0_objprops_Relative_2**^**nd**^ **axis length**	**0.015**
	Frangi_objprops_mean intensity	0,610
f2000	**Pyrad_log_sigma_2mm_3D_glcm_Idn**	**0.006**
	**Pyrad_log_sigma_1mm_3D_glcm_Idn**	**0.006**
	**Pyrad_log_sigma_3mm_3D_glcm_Idn**	**0.006**
Ds2000	**LBP_skewness**	**<0.001**
	**Pyrad_wavelet_HLH_glszm_SmallAreaHighGrayLevelEmphasis**	**<0.001**
	**Hessian_0.025_15.0_objprops_Orientation_SD**	**0.009**
Ds4000	Pyrad_wavelet_HHH_glszm_SmallAreaHighGrayLevelEmphasis	0.097
	**Pyrad_wavelet_HHL_glszm_SizeZoneNonUniformity**	**<0.001**
	**Pyrad_wavelet_HLH_glszm_SmallAreaHighGrayLevelEmphasis**	**<0.001**
Df2000	**Pyrad_wavelet_LLH_glszm_SizeZoneNonUniformity**	**<0.001**
	**Pyrad_wavelet_LLL_glszm_SizeZoneNonUniformity**	**<0.001**
	**Pyrad_wavelet_HLH_glszm_SizeZoneNonUniformity**	**<0.001**
Df4000	**Gabor_f1.00_d2_k4_skewness**	**<0.001**
	Pyrad InterquartileRange	0,0701
	Pyrad wavelet LLL InterquartileRange	0.069

Pyrad: Pyrad package radiomics

Frangi: Frangi filtered intensity values for radiomics

Hessian: Hessian filtered intensity values for radiomics

LPB: Local binary pattern intensity values for radiomics

Gabor: Gabor filtered intensity values for radiomics

wavelet: wavelet compoments, L = Lowpass, H = highpass

log: Laplacian edge enhancement filter

glcm: Gray Level Co-occurrence Matrix

glszm: Gray Level Size Zone

objprops: object properties of non-zero values after filtering

## Discussion

In prior preclinical studies spin lock imaging demonstrated promising results for non-invasive characterization of brain gliomas. Gliomas tend to have high signal on T2 based sequences. The hyperintensity on T2 based sequences (including fluid-attenuated inversion-recovery, FLAIR) is due to prolongation of transverse relaxation times (T2 relaxation time) mainly caused by the increase in tissue water content and ultrastructural changes. The areas of haemosiderin appear as foci of signal dropout. However, anatomical (T2-/T1-/FLAIR weighted imaging) have limited potential for predication of IDH mutation and 1p/19q codeletion [12]. Accurate detection of IDH mutation and 1p/19q codeletion is important for risk stratification since these markers have predicative power for longer survival and distinguishing oligodendrogliomas from diffuse astrocytomas, respectively [2]. Thus, development of novel imaging methods for non-invasive detection IDH mutation and 1p/19q codeletion could offer new possibilities for patients’ risk management and tailored treatment.

We used whole tumor FLAIR lesion segmentation, histogram and radiomic analysis to differentiate gliomas according to their IDH gene and 1p/19q chromosomal status. Following correction for effects of age and gender, at least one statistical descriptor per each spin lock imaging method and various radiomic features were able to significantly differentiate between IDH and 1p/19q codeletion groups following correction for multiple comparison. Although we demonstrated statistical significance, the clinical implications of these findings remain to the explored.

Our study has a number of limitations. Only 22 patients were included and further studies are need to explore our preliminary findings in a larger study cohort. The current study did not aim to explore relaxation mechanisms of T_1ρadiab_, and T_RAFF2_. Different relaxation methods, including dipolar interactions, diffusion, exchange relaxation pathways, can all contribute to various degree to T_RAFF2_, T_1ρcw_, T_1ρadiab_, and T_2ρadiab_ [3, 6, 13–15]. Although this study demonstrated clinical significance for several variables, the clinical relevance of the findings and impact on patient outcome is left to be explored in larger clinical trials. Three patients withdrawn from the trial precluding the use of histopathological material for the analyses which may have introduced a bias and affect the completeness of the study. Finally, this study used only a limited number of radiomics due to the small sample size and future studies using large number of radiomics and various machine learning methods are warranted [16–18]. Please note, the interpretability of these radionics features and their direct biological or clinical relevance is beyond the scope of this study.

## Conclusion

Our clinical trial demonstrated feasibility of quantitative T_RAFF2_, T_1ρcw_, T_1ρadiab_, and T_2ρadiab_ imaging of human gliomas using a clinical 3T MRI scanner. Our initial results indicate the potential of these methods to improve non-invasive glioma characterization.

## Supporting information

S1 Checklist Human participants research checklist. (DOCX)

S2 Checklist Reporting checklist for diagnostic test accuracy study. (DOCX)

S1 File (DOCX)
