# Supplementary material for: Relaxation Along a Fictitious Field, continuous wave T1rho, adiabatic T1rho and adiabatic T2rho imaging of human gliomas at 3T: A feasibility study
Source: PLoS One. 2024 Apr 1;19(4):e0296958. doi: 10.1371/journal.pone.0296958 (PMC10984536; doi:10.1371/journal.pone.0296958)
Supplement: S1 File — (DOCX) [file pone.0296958.s003.docx]

**Relaxation Along a Fictitious Field, continuous wave T1rho, adiabatic T1rho and adiabatic T2rho imaging of human gliomas at 3T: a feasibility study**

**Supporting Material**

**Supporting Material Table 1**

Region of Interest (ROI) descriptive statistics differences between IDH-mutated (IDH mut) vs IDH-wild-type (IDH wt) gliomas. For MRI variables, Variable~Group+Age+Sex-1 model was used to correct for effects of age and sex. * FDR-corrected p-value < 0.05, ** p-value < 0.01, *** p-value < 0.001.

|  | **Measure** | **Group** | **IDH all** | **IDH mut** | **IDH wild type** |  |  |
| --- | --- | --- | --- | --- | --- | --- | --- |
|  | N | All | 22 | 10 | 12 |  |  |
|  | N(lesions) | All | 83 | 41 | 42 |  |  |
| **Significance** | **Measure** | **Group** | **IDH all** | **IDH mut** | **IDH wild type** | **p-value** | **FDR-corrected p-value** |
| *** | Age | All | 48.52±14.64 | 42.80±16.42 | 54.10±10.04 | 0.00 | 0.00 |
|  |  |  | **All** | **Male** | **Female** | **p-value (proportions)** | **FDR-corrected p-value** |
| *** | Lesions | IDH mut | 41 | 18 | 23 | 0.00 | 0.00 |
| *** |  | IDH wt | 42 | 33 | 9 | 0.00 | 0.00 |
| **Significance** | **Modality** | **MRI Variable** | **IDH all** | **IDH mut** | **IDH wild type** | **p-value** | **FDR-corrected p-value** |
|  | T2W | mean | 976.24±226.65 | 1023.07±177.71 | 930.52±260.00 | 0.35 | 0.40 |
|  | T2W | median | 963.42±232.28 | 1009.63±190.88 | 918.32±261.04 | 0.35 | 0.40 |
|  | T2W | 25percentile | 872.28±183.49 | 913.42±130.17 | 832.11±217.86 | 0.35 | 0.40 |
|  | T2W | 75percentile | 1067.24±291.42 | 1117.60±249.49 | 1018.07±322.66 | 0.41 | 0.45 |
| *** | T2W | skewness | 0.77±1.08 | 0.79±1.03 | 0.75±1.14 | 0.00 | 0.00 |
| *** | T2W | kurtosis | 2.97±4.84 | 2.37±3.53 | 3.56±5.82 | 0.00 | 0.00 |
|  | T2W | SD | 155.91±94.73 | 158.20±83.12 | 153.68±105.82 | 0.31 | 0.40 |
| *** | T2HS | mean | 51.73±29.82 | 51.08±29.41 | 52.37±30.57 | 0.00 | 0.00 |
| *** | T2HS | median | 50.93±33.58 | 51.13±34.39 | 50.72±33.18 | 0.00 | 0.00 |
|  | T2HS | 25percentile | 38.81±32.96 | 39.00±34.34 | 38.62±31.97 | 0.20 | 0.28 |
| *** | T2HS | 75percentile | 62.95±36.67 | 61.71±33.92 | 64.16±39.55 | 0.00 | 0.00 |
| *** | T2HS | skewness | 0.66±4.40 | 1.33±5.91 | 0.00±1.92 | 0.00 | 0.00 |
| *** | T2HS | kurtosis | 20.13±111.29 | 37.32±157.17 | 3.35±9.28 | 0.00 | 0.00 |
| *** | T2HS | SD | 20.31±15.03 | 18.57±13.56 | 22.02±16.32 | 0.00 | 0.00 |
| *** | T1HS | mean | 189.11±149.76 | 159.12±101.16 | 218.39±181.93 | 0.00 | 0.00 |
| *** | T1HS | median | 168.33±153.41 | 147.58±101.92 | 188.58±189.97 | 0.00 | 0.00 |
| *** | T1HS | 25percentile | 119.08±119.30 | 104.90±95.25 | 132.92±138.62 | 0.00 | 0.00 |
| *** | T1HS | 75percentile | 232.57±216.45 | 189.40±117.97 | 274.72±276.48 | 0.00 | 0.00 |
| *** | T1HS | skewness | 1.76±2.87 | 2.31±3.44 | 1.23±2.08 | 0.00 | 0.00 |
| *** | T1HS | kurtosis | 13.68±42.70 | 20.99±59.22 | 6.54±10.90 | 0.00 | 0.00 |
| *** | T1HS | SD | 112.93±99.94 | 87.18±70.54 | 138.07±117.52 | 0.00 | 0.00 |
| *** | T1CW | mean | 84.74±67.91 | 73.99±52.69 | 95.24±79.28 | 0.00 | 0.00 |
| * | T1CW | median | 64.89±47.68 | 66.16±47.31 | 63.65±48.59 | 0.01 | 0.01 |
|  | T1CW | 25percentile | 46.48±41.23 | 46.62±42.05 | 46.34±40.91 | 0.35 | 0.40 |
| *** | T1CW | 75percentile | 93.27±78.22 | 86.29±60.51 | 100.09±92.57 | 0.00 | 0.00 |
|  | T1CW | skewness | 2.75±3.51 | 3.23±3.83 | 2.28±3.14 | 0.46 | 0.49 |
| * | T1CW | kurtosis | 26.21±52.56 | 35.58±67.90 | 17.06±29.23 | 0.01 | 0.02 |
| *** | T1CW | SD | 75.91±90.09 | 51.56±54.60 | 99.67±110.24 | 0.00 | 0.00 |
| *** | RAFF | mean | 141.58±113.01 | 129.70±92.59 | 153.17±130.01 | 0.00 | 0.00 |
| *** | RAFF | median | 118.57±104.35 | 116.85±83.14 | 120.26±122.59 | 0.00 | 0.00 |
| *** | RAFF | 25percentile | 83.84±81.61 | 81.60±73.40 | 86.02±89.76 | 0.00 | 0.00 |
| *** | RAFF | 75percentile | 167.85±161.89 | 154.21±109.21 | 181.16±201.04 | 0.00 | 0.00 |
|  | RAFF | skewness | 2.21±3.25 | 2.68±3.82 | 1.75±2.54 | 0.40 | 0.44 |
| *** | RAFF | kurtosis | 18.63±47.71 | 26.36±65.01 | 11.08±17.60 | 0.00 | 0.00 |
| *** | RAFF | SD | 100.35±98.59 | 79.64±73.29 | 120.57±115.55 | 0.00 | 0.00 |
|  | FLAIR | mean | 1152.30±321.15 | 1242.87±295.79 | 1063.88±323.58 | 0.23 | 0.32 |
|  | FLAIR | median | 1125.62±317.02 | 1216.63±299.26 | 1036.78±311.88 | 0.12 | 0.18 |
| * | FLAIR | 25percentile | 1024.05±274.49 | 1105.59±265.59 | 944.45±262.16 | 0.01 | 0.02 |
|  | FLAIR | 75percentile | 1257.15±385.83 | 1354.98±347.05 | 1161.64±401.64 | 0.35 | 0.40 |
| *** | FLAIR | skewness | 1.00±1.19 | 0.89±1.07 | 1.11±1.30 | 0.00 | 0.00 |
|  | FLAIR | kurtosis | 3.80±10.24 | 2.66±7.88 | 4.91±12.12 | 0.13 | 0.18 |
|  | FLAIR | SD | 176.91±118.28 | 190.16±110.49 | 163.98±125.39 | 0.09 | 0.13 |
| *** | f2000 | mean | 0.21±0.13 | 0.19±0.13 | 0.22±0.13 | 0.00 | 0.00 |
| *** | f2000 | median | 0.19±0.15 | 0.18±0.14 | 0.19±0.15 | 0.00 | 0.00 |
| *** | f2000 | 25percentile | 0.12±0.12 | 0.11±0.12 | 0.13±0.11 | 0.00 | 0.00 |
| *** | f2000 | 75percentile | 0.27±0.17 | 0.25±0.16 | 0.28±0.17 | 0.00 | 0.00 |
|  | f2000 | skewness | 1.77±4.00 | 2.02±5.18 | 1.52±2.39 | 0.56 | 0.58 |
| *** | f2000 | kurtosis | 20.45±121.18 | 31.24±168.58 | 9.93±37.58 | 0.00 | 0.00 |
| *** | f2000 | SD | 0.14±0.09 | 0.12±0.07 | 0.16±0.10 | 0.00 | 0.00 |
| *** | Ds2000 | mean | 0.02±0.04 | 0.01±0.04 | 0.03±0.05 | 0.00 | 0.00 |
|  | Ds2000 | median | 0.00±0.00 | 0.00±0.00 | 0.00±0.00 | 0.35 | 0.40 |
|  | Ds2000 | 25percentile | 0.00±0.00 | 0.00±0.00 | 0.00±0.00 | 0.35 | 0.40 |
|  | Ds2000 | 75percentile | 0.00±0.00 | 0.00±0.00 | 0.00±0.00 | 0.35 | 0.40 |
|  | Ds2000 | skewness | 7.13±31.42 | 4.61±8.46 | 9.59±43.50 | 0.55 | 0.57 |
|  | Ds2000 | kurtosis | 1038.14±8871.93 | 89.89±274.74 | 1963.82±12472.84 | 0.77 | 0.77 |
| *** | Ds2000 | SD | 0.07±0.11 | 0.06±0.09 | 0.08±0.12 | 0.00 | 0.00 |
| *** | Ds4000 | mean | 0.02±0.04 | 0.01±0.04 | 0.03±0.05 | 0.00 | 0.00 |
|  | Ds4000 | median | 0.00±0.00 | 0.00±0.00 | 0.00±0.00 | 0.35 | 0.40 |
| * | Ds4000 | 25percentile | 0.00±0.00 | 0.00±0.00 | 0.00±0.00 | 0.01 | 0.02 |
|  | Ds4000 | 75percentile | 0.00±0.00 | 0.00±0.00 | 0.00±0.00 | 0.35 | 0.40 |
|  | Ds4000 | skewness | 7.08±31.90 | 4.44±8.55 | 9.65±44.16 | 0.55 | 0.57 |
|  | Ds4000 | kurtosis | 1057.36±9046.31 | 89.96±274.72 | 2001.72±12718.04 | 0.77 | 0.77 |
| *** | Ds4000 | SD | 0.07±0.11 | 0.06±0.09 | 0.08±0.12 | 0.00 | 0.00 |
| *** | Df2000 | mean | 0.05±0.10 | 0.03±0.06 | 0.06±0.12 | 0.00 | 0.00 |
| *** | Df2000 | median | 0.03±0.11 | 0.02±0.04 | 0.05±0.15 | 0.00 | 0.00 |
| *** | Df2000 | 25percentile | 0.01±0.04 | 0.01±0.02 | 0.01±0.05 | 0.00 | 0.00 |
| *** | Df2000 | 75percentile | 0.06±0.14 | 0.04±0.08 | 0.09±0.18 | 0.00 | 0.00 |
|  | Df2000 | skewness | 4.17±5.59 | 4.54±5.59 | 3.81±5.64 | 0.35 | 0.40 |
|  | Df2000 | kurtosis | 57.51±182.38 | 62.96±176.26 | 52.19±190.15 | 0.35 | 0.40 |
| *** | Df2000 | SD | 0.06±0.09 | 0.04±0.06 | 0.07±0.10 | 0.00 | 0.00 |
| ** | Df4000 | mean | 0.14±0.16 | 0.12±0.15 | 0.15±0.17 | 0.00 | 0.01 |
| * | Df4000 | median | 0.11±0.16 | 0.11±0.16 | 0.12±0.17 | 0.03 | 0.05 |
| *** | Df4000 | 25percentile | 0.05±0.12 | 0.05±0.09 | 0.06±0.14 | 0.00 | 0.00 |
| * | Df4000 | 75percentile | 0.19±0.25 | 0.17±0.23 | 0.22±0.27 | 0.01 | 0.01 |
|  | Df4000 | skewness | 2.36±4.24 | 2.67±5.13 | 2.06±3.17 | 0.40 | 0.44 |
| *** | Df4000 | kurtosis | 25.18±124.26 | 34.81±168.35 | 15.78±55.17 | 0.00 | 0.00 |
| *** | Df4000 | SD | 0.11±0.11 | 0.10±0.10 | 0.13±0.12 | 0.00 | 0.00 |

**Supporting Material Table 2**

Region of Interest (ROI) descriptive statistics differences between astrocytic gliomas without 1p/19q codeletion (A) oligodendroglioma IDH-mutant and 1p/19q co-deleted (O). For MRI variables, Variable~Group+Age+Sex-1 model was used to correct for effects of age and sex. * FDR-corrected p-value < 0.05, ** p-value < 0.01, *** p-value < 0.001.

|  | **Measure** | **Group** | **All** | **A** | **O** |  |  |
| --- | --- | --- | --- | --- | --- | --- | --- |
|  | N | All | 9 | 4 | 5 |  |  |
|  | N(lesions) | All | 38 | 12 | 26 |  |  |
| **Significance** | **Measure** | **Group** | **All** | **A** | **O** | **p-value** | **FDR-corrected p-value** |
|  | Age | All | 41.45±16.30 | 40.92±14.13 | 41.69±17.47 | 0.67 | 0.77 |
|  |  |  | **All** | **Male** | **Female** | **p-value (proportions)** | **FDR-corrected p-value** |
|  | Lesions | A | 12 | 7 | 5 | 0.37 | 0.45 |
|  |  | O | 26 | 11 | 15 | 0.37 | 0.45 |
| **Significance** | **Modality** | **Statistic** | **All** | **A** | **O** | **p-value** | **FDR-corrected p-value** |
| ** | T2W | mean | 1025.79±182.84 | 1023.76±180.65 | 1026.73±187.38 | 0.00 | 0.00 |
| ** | T2W | median | 1012.22±196.09 | 998.50±182.84 | 1018.55±205.11 | 0.00 | 0.00 |
| ** | T2W | 25percentile | 913.07±133.86 | 899.47±132.46 | 919.34±136.63 | 0.00 | 0.00 |
| ** | T2W | 75percentile | 1123.49±255.58 | 1127.97±243.63 | 1121.43±265.60 | 0.00 | 0.00 |
| *** | T2W | skewness | 0.79±1.00 | 0.78±0.81 | 0.80±1.08 | 0.00 | 0.00 |
| *** | T2W | kurtosis | 2.14±3.33 | 1.39±2.84 | 2.48±3.54 | 0.00 | 0.00 |
|  | T2W | SD | 160.74±85.79 | 173.27±77.28 | 154.96±90.30 | 0.79 | 0.86 |
| *** | T2HS | mean | 49.77±29.95 | 47.10±28.57 | 51.00±31.04 | 0.00 | 0.00 |
| *** | T2HS | median | 49.43±35.14 | 47.82±34.97 | 50.17±35.88 | 0.00 | 0.00 |
| *** | T2HS | 25percentile | 38.20±34.24 | 33.45±34.15 | 40.39±34.72 | 0.00 | 0.00 |
| *** | T2HS | 75percentile | 60.55±34.96 | 58.75±35.77 | 61.38±35.26 | 0.00 | 0.00 |
|  | T2HS | skewness | 1.39±6.14 | 2.58±9.09 | 0.83±4.29 | 0.20 | 0.25 |
|  | T2HS | kurtosis | 40.09±163.09 | 82.61±273.36 | 20.47±72.24 | 0.20 | 0.25 |
| *** | T2HS | SD | 18.87±13.51 | 21.31±14.17 | 17.74±13.33 | 0.00 | 0.00 |
| ** | T1HS | mean | 155.61±103.68 | 153.86±98.44 | 156.42±107.90 | 0.00 | 0.00 |
| *** | T1HS | median | 142.82±104.05 | 139.11±104.82 | 144.54±105.73 | 0.00 | 0.00 |
| *** | T1HS | 25percentile | 102.48±94.66 | 92.77±97.31 | 106.96±95.02 | 0.00 | 0.00 |
| ** | T1HS | 75percentile | 186.19±121.52 | 187.41±121.84 | 185.64±123.78 | 0.01 | 0.01 |
| ** | T1HS | skewness | 2.30±3.56 | 2.10±2.00 | 2.39±4.12 | 0.00 | 0.00 |
| ** | T1HS | kurtosis | 21.87±61.46 | 13.98±19.80 | 25.50±73.31 | 0.00 | 0.00 |
|  | T1HS | SD | 87.84±71.21 | 95.69±77.20 | 84.21±69.57 | 0.06 | 0.08 |
|  | T1CW | mean | 73.18±54.58 | 67.38±42.94 | 75.86±59.78 | 0.06 | 0.08 |
| ** | T1CW | median | 64.75±48.81 | 58.67±44.05 | 67.56±51.44 | 0.00 | 0.00 |
| *** | T1CW | 25percentile | 45.81±42.13 | 38.98±40.27 | 48.96±43.36 | 0.00 | 0.00 |
| * | T1CW | 75percentile | 85.90±62.80 | 77.62±49.75 | 89.72±68.56 | 0.02 | 0.03 |
| *** | T1CW | skewness | 3.18±3.93 | 3.01±2.53 | 3.25±4.47 | 0.00 | 0.00 |
| *** | T1CW | kurtosis | 36.15±70.36 | 31.13±58.98 | 38.47±76.02 | 0.00 | 0.00 |
|  | T1CW | SD | 51.92±54.97 | 53.58±58.61 | 51.16±54.39 | 0.86 | 0.86 |
|  | RAFF | mean | 128.24±95.69 | 118.21±74.97 | 132.87±104.92 | 0.06 | 0.08 |
| ** | RAFF | median | 114.28±85.59 | 106.66±81.74 | 117.80±88.66 | 0.00 | 0.00 |
| *** | RAFF | 25percentile | 80.13±73.32 | 71.02±73.92 | 84.34±74.12 | 0.00 | 0.00 |
| * | RAFF | 75percentile | 153.42±113.18 | 141.86±93.06 | 158.76±122.69 | 0.02 | 0.03 |
| *** | RAFF | skewness | 2.60±3.92 | 2.57±2.13 | 2.60±4.56 | 0.00 | 0.00 |
| *** | RAFF | kurtosis | 26.20±67.31 | 18.71±27.24 | 29.67±79.62 | 0.00 | 0.00 |
|  | RAFF | SD | 80.70±74.22 | 80.15±61.20 | 80.95±80.65 | 0.86 | 0.86 |
| *** | FLAIR | mean | 1256.28±302.95 | 1260.09±340.42 | 1254.53±291.26 | 0.00 | 0.00 |
| *** | FLAIR | median | 1229.55±306.88 | 1233.05±333.85 | 1227.93±300.55 | 0.00 | 0.00 |
| *** | FLAIR | 25percentile | 1114.85±273.84 | 1103.11±287.21 | 1120.26±273.11 | 0.00 | 0.00 |
| *** | FLAIR | 75percentile | 1373.31±353.19 | 1389.10±397.00 | 1366.01±339.26 | 0.00 | 0.00 |
| *** | FLAIR | skewness | 0.69±0.51 | 0.58±0.39 | 0.74±0.56 | 0.00 | 0.00 |
| *** | FLAIR | kurtosis | 1.06±1.63 | 0.38±0.85 | 1.37±1.81 | 0.00 | 0.00 |
|  | FLAIR | SD | 192.87±113.35 | 202.09±97.45 | 188.61±121.55 | 0.06 | 0.08 |
| * | f2000 | mean | 0.19±0.13 | 0.19±0.11 | 0.20±0.14 | 0.02 | 0.03 |
| * | f2000 | median | 0.18±0.15 | 0.17±0.13 | 0.18±0.15 | 0.01 | 0.02 |
|  | f2000 | 25percentile | 0.11±0.12 | 0.11±0.11 | 0.12±0.13 | 0.06 | 0.08 |
| ** | f2000 | 75percentile | 0.25±0.17 | 0.23±0.14 | 0.26±0.18 | 0.00 | 0.00 |
| ** | f2000 | skewness | 2.10±5.37 | 1.38±1.53 | 2.43±6.43 | 0.00 | 0.00 |
|  | f2000 | kurtosis | 33.39±175.09 | 4.63±7.30 | 46.66±211.58 | 0.16 | 0.21 |
| ** | f2000 | SD | 0.13±0.08 | 0.13±0.08 | 0.13±0.07 | 0.01 | 0.01 |
|  | Ds2000 | mean | 0.01±0.04 | 0.01±0.03 | 0.01±0.04 | 0.13 | 0.18 |
| ** | Ds2000 | median | 0.00±0.00 | 0.00±0.00 | 0.00±0.00 | 0.00 | 0.00 |
| * | Ds2000 | 25percentile | 0.00±0.00 | 0.00±0.00 | 0.00±0.00 | 0.02 | 0.03 |
| ** | Ds2000 | 75percentile | 0.00±0.00 | 0.00±0.00 | 0.00±0.00 | 0.00 | 0.00 |
| *** | Ds2000 | skewness | 4.73±8.74 | 1.51±2.89 | 6.21±10.11 | 0.00 | 0.00 |
| ** | Ds2000 | kurtosis | 95.39±284.84 | 8.87±13.36 | 135.33±338.74 | 0.00 | 0.00 |
|  | Ds2000 | SD | 0.06±0.09 | 0.05±0.10 | 0.06±0.09 | 0.86 | 0.86 |
|  | Ds4000 | mean | 0.01±0.04 | 0.01±0.03 | 0.01±0.04 | 0.13 | 0.18 |
| *** | Ds4000 | median | 0.00±0.00 | 0.00±0.00 | 0.00±0.00 | 0.00 | 0.00 |
| *** | Ds4000 | 25percentile | 0.00±0.00 | 0.00±0.00 | 0.00±0.00 | 0.00 | 0.00 |
| *** | Ds4000 | 75percentile | 0.00±0.00 | 0.00±0.00 | 0.00±0.00 | 0.00 | 0.00 |
| *** | Ds4000 | skewness | 4.57±8.83 | 1.34±2.93 | 6.06±10.21 | 0.00 | 0.00 |
| ** | Ds4000 | kurtosis | 95.58±284.78 | 8.54±13.46 | 135.76±338.58 | 0.00 | 0.00 |
|  | Ds4000 | SD | 0.06±0.09 | 0.05±0.10 | 0.06±0.09 | 0.86 | 0.86 |
|  | Df2000 | mean | 0.03±0.06 | 0.01±0.01 | 0.04±0.08 | 0.46 | 0.53 |
|  | Df2000 | median | 0.02±0.04 | 0.01±0.01 | 0.02±0.05 | 0.30 | 0.37 |
|  | Df2000 | 25percentile | 0.01±0.02 | 0.00±0.00 | 0.01±0.03 | 0.30 | 0.37 |
|  | Df2000 | 75percentile | 0.04±0.09 | 0.02±0.02 | 0.05±0.10 | 0.46 | 0.53 |
| ** | Df2000 | skewness | 4.47±5.72 | 5.04±4.05 | 4.21±6.41 | 0.00 | 0.01 |
|  | Df2000 | kurtosis | 63.86±182.72 | 62.05±107.26 | 64.69±210.59 | 0.17 | 0.22 |
|  | Df2000 | SD | 0.04±0.06 | 0.02±0.02 | 0.05±0.07 | 0.86 | 0.86 |
|  | Df4000 | mean | 0.13±0.16 | 0.09±0.08 | 0.14±0.18 | 0.86 | 0.86 |
|  | Df4000 | median | 0.11±0.17 | 0.07±0.07 | 0.13±0.20 | 0.86 | 0.86 |
|  | Df4000 | 25percentile | 0.05±0.09 | 0.04±0.04 | 0.06±0.10 | 0.86 | 0.86 |
|  | Df4000 | 75percentile | 0.18±0.24 | 0.11±0.10 | 0.21±0.27 | 0.86 | 0.86 |
| ** | Df4000 | skewness | 2.66±5.32 | 2.44±1.55 | 2.77±6.39 | 0.00 | 0.00 |
|  | Df4000 | kurtosis | 36.24±174.93 | 11.36±10.62 | 47.72±211.67 | 0.16 | 0.21 |

**Supporting Material Table 3**

Spearman correlation analysis between Ki-67 index and MRI relaxation time constants in brain glioma patients, using median value inside ROI. The radiomic variables were evaluated with model Ki67~Variable+Age+Sex-1. FLAIR mean, median and 25% percentile were found to have statistically significant correlation after correction for multiple comparisons over evaluations on basic statistical descriptors. * FDR-corrected p-value < 0.05, ** p-value < 0.01, *** p-value < 0.001.

| **Significance** | **Measure** | **Pearson r** | **p-value** | **FDR-corrected p-value** |
| --- | --- | --- | --- | --- |
| *** | Age | 0.360 | <0.001 | <0.001 |
|  | Sex | 0.113 | 0.094 | 0.972 |
| **Significance** | **Radiomic Variable** | **beta** | **p-value** | **FDR-corrected p-value** |
|  | T2W_mean | -0.078 | 0.424 | 0.972 |
|  | T2W_median | -0.083 | 0.396 | 0.972 |
|  | T2W_25percentile | -0.129 | 0.185 | 0.972 |
|  | T2W_75percentile | -0.036 | 0.711 | 0.972 |
|  | T2W_skewness | -0.004 | 0.970 | 0.982 |
|  | T2W_kurtosis | 0.095 | 0.332 | 0.972 |
|  | T2W_SD | 0.073 | 0.454 | 0.972 |
|  | T2HS_mean | 0.001 | 0.988 | 0.988 |
|  | T2HS_median | 0.025 | 0.801 | 0.972 |
|  | T2HS_25percentile | -0.024 | 0.809 | 0.972 |
|  | T2HS_75percentile | 0.028 | 0.772 | 0.972 |
|  | T2HS_skewness | -0.106 | 0.278 | 0.972 |
|  | T2HS_kurtosis | -0.102 | 0.296 | 0.972 |
|  | T2HS_SD | 0.027 | 0.785 | 0.972 |
|  | T1HS_mean | 0.060 | 0.538 | 0.972 |
|  | T1HS_median | 0.064 | 0.514 | 0.972 |
|  | T1HS_25percentile | 0.041 | 0.673 | 0.972 |
|  | T1HS_75percentile | 0.074 | 0.447 | 0.972 |
|  | T1HS_skewness | -0.155 | 0.110 | 0.972 |
|  | T1HS_kurtosis | -0.130 | 0.181 | 0.972 |
|  | T1HS_SD | 0.050 | 0.607 | 0.972 |
|  | T1CW_mean | 0.034 | 0.729 | 0.972 |
|  | T1CW_median | 0.039 | 0.687 | 0.972 |
|  | T1CW_25percentile | -0.012 | 0.906 | 0.982 |
|  | T1CW_75percentile | 0.017 | 0.860 | 0.974 |
|  | T1CW_skewness | -0.148 | 0.128 | 0.972 |
|  | T1CW_kurtosis | -0.158 | 0.105 | 0.972 |
|  | T1CW_SD | 0.044 | 0.652 | 0.972 |
|  | RAFF_mean | 0.005 | 0.963 | 0.982 |
|  | RAFF_median | 0.022 | 0.825 | 0.972 |
|  | RAFF_25percentile | -0.015 | 0.875 | 0.974 |
|  | RAFF_75percentile | -0.007 | 0.945 | 0.982 |
|  | RAFF_skewness | -0.143 | 0.141 | 0.972 |
|  | RAFF_kurtosis | -0.130 | 0.182 | 0.972 |
|  | RAFF_SD | 0.018 | 0.853 | 0.974 |
| ** | FLAIR_mean | -0.338 | <0.001 | 0.007 |
| ** | FLAIR_median | -0.346 | <0.001 | 0.007 |
| ** | FLAIR_25percentile | -0.387 | <0.001 | 0.002 |
|  | FLAIR_75percentile | -0.277 | 0.004 | 0.061 |
|  | FLAIR_skewness | 0.092 | 0.347 | 0.972 |
|  | FLAIR_kurtosis | 0.115 | 0.236 | 0.972 |
|  | FLAIR_SD | -0.045 | 0.648 | 0.972 |
|  | f2000_mean | 0.005 | 0.962 | 0.982 |
|  | f2000_median | 0.010 | 0.918 | 0.982 |
|  | f2000_25percentile | 0.022 | 0.821 | 0.972 |
|  | f2000_75percentile | 0.034 | 0.728 | 0.972 |
|  | f2000_skewness | -0.099 | 0.308 | 0.972 |
|  | f2000_kurtosis | -0.069 | 0.479 | 0.972 |
|  | f2000_SD | -0.056 | 0.569 | 0.972 |
|  | Ds2000_mean | -0.104 | 0.285 | 0.972 |
|  | Ds2000_median | -0.041 | 0.675 | 0.972 |
|  | Ds2000_25percentile | -0.024 | 0.810 | 0.972 |
|  | Ds2000_75percentile | -0.033 | 0.738 | 0.972 |
|  | Ds2000_skewness | 0.058 | 0.555 | 0.972 |
|  | Ds2000_kurtosis | 0.074 | 0.448 | 0.972 |
|  | Ds2000_SD | -0.089 | 0.363 | 0.972 |
|  | Ds4000_mean | -0.105 | 0.284 | 0.972 |
|  | Ds4000_median | -0.077 | 0.431 | 0.972 |
|  | Ds4000_25percentile | -0.048 | 0.627 | 0.972 |
|  | Ds4000_75percentile | -0.080 | 0.412 | 0.972 |
|  | Ds4000_skewness | 0.057 | 0.559 | 0.972 |
|  | Ds4000_kurtosis | 0.074 | 0.447 | 0.972 |
|  | Ds4000_SD | -0.089 | 0.363 | 0.972 |
|  | Df2000_mean | 0.059 | 0.546 | 0.972 |
|  | Df2000_median | -0.016 | 0.868 | 0.974 |
|  | Df2000_25percentile | -0.026 | 0.794 | 0.972 |
|  | Df2000_75percentile | 0.031 | 0.753 | 0.972 |
|  | Df2000_skewness | -0.122 | 0.212 | 0.972 |
|  | Df2000_kurtosis | -0.070 | 0.471 | 0.972 |
|  | Df2000_SD | 0.154 | 0.112 | 0.972 |
|  | Df4000_mean | 0.061 | 0.533 | 0.972 |
|  | Df4000_median | 0.005 | 0.957 | 0.982 |
|  | Df4000_25percentile | -0.024 | 0.807 | 0.972 |
